# Quantitative MRI of dorsal root ganglion alterations in neurofibromatosis type 1 patients with or without pain

**DOI:** 10.1186/s41747-025-00594-x

**Published:** 2025-05-28

**Authors:** Magnus Schindehütte, Eva Meller, Thomas Kampf, Florian Hessenauer, Nurcan Üçeyler, György Homola, Heike L. Rittner, Cordula Matthies, Mirko Pham, Simon Weiner

**Affiliations:** 1 Department of Neuroradiology, University Hospital Würzburg, Würzburg, Germany; 2 Department of Neurosurgery, University Hospital Würzburg, Würzburg, Germany; 3 Würzburg Centre for Neurofibromatosis, Centre for Rare Diseases, University Hospital Würzburg, Würzburg, Germany; 4 Department of Neurology, University Hospital Würzburg, Würzburg, Germany; 5 Department of Anesthesiology, Intensive Care, Emergency and Pain Medicine, Centre for Interdisciplinary Pain Medicine, University Hospital Würzburg, Würzburg, Germany

**Keywords:** Biomarkers, Chronic pain, Ganglia (spinal), Magnetic resonance imaging, Neurofibromatosis 1

## Abstract

**Background:**

Neurofibromatosis type 1 (NF1) is a genetic disorder characterised by skin and nervous system anomalies, primarily involving glial cells and nerve tumours. Pain, particularly chronic pain, is a significant but often overlooked symptom in NF1 patients, affecting their health-related quality of life. The dorsal root ganglion (DRG) is essential for pain signal transmission, yet *in vivo* studies of DRG in NF1 patients are lacking.

**Methods:**

This prospective study included 20 NF1 patients (8 with neuropathic pain) and 28 healthy controls. Magnetic resonance imaging (MRI) scans of lumbosacral DRG (L5 + S1) were performed using a 3-T scanner. Quantitative MRI techniques were applied to assess DRG volume, T2 relaxation time, and proton density (PD). Statistical analyses compared NF1 patients and controls, and NF1 patients with and without pain.

**Results:**

NF1 patients had a significantly larger DRG volume and higher quantitative T2 and PD values compared to controls. Furthermore, DRG PD was significantly higher in NF1 patients with neuropathic pain than in those without pain. Receiver operator characteristic curve analysis identified DRG PD as the best discriminator of pain in NF1 patients, with an area under the curve of 0.84, indicating relevant and useful discriminatory power.

**Conclusion:**

NF1 patients showed objective macrostructural and microstructural DRG injury changes using dedicated DRG MRI, discriminating neuropathic pain status from non-pain status at the disease-symptom group level. These findings highlight the potential of DRG MRI to quantify DRG pathology *in vivo* and to determine the risk of functional pain status by imaging.

**Relevance statement:**

The identification of structural and microstructural changes of the DRG by quantitative MRI provides a novel *in vivo* biomarker for understanding neuropathic pain mechanisms, pain risk assessment and treatment monitoring in NF1.

**Key Points:**

- Dorsal root ganglia (DRG) in NF1 are enlarged by 176.3% in MRI.
- In quantitative MRI of DRG NF1, T2 relaxation time is increased by 22.9% and PD by 8.4%.
- DRG PD can distinguish a painful from a non-painful NF1 phenotype.

**Graphical Abstract:**

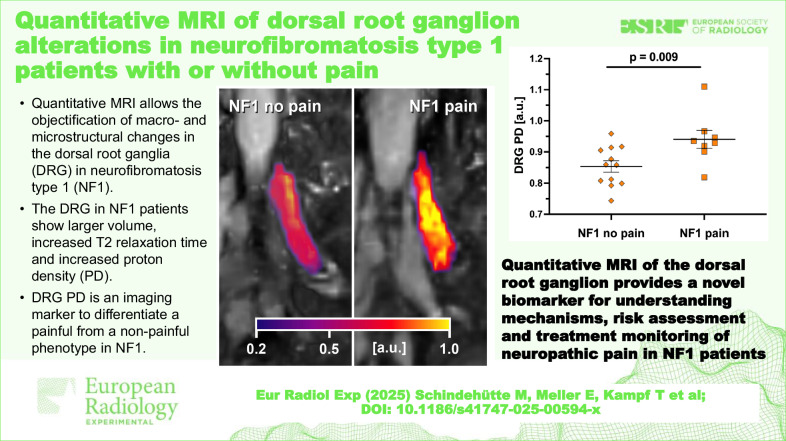

## Background

Neurofibromatosis type 1 (NF1) is an inherited disorder of the skin and nervous system characterised by the formation of nerve tumours, particularly neurofibromas [1]. The disease is caused by mutations in the NF1 gene, a tumour suppressor gene located on chromosome 17 [2–4]. It encodes for the protein neurofibromin, which is involved in several cell-signalling pathways, regulating proliferation and migration, cytoskeletal dynamics, neurite outgrowth, dendritic-spine density, and dopamine levels [5]. Neurofibromin depletion leads to abnormal cell growth [2]. The clinical manifestations of NF1 are highly variable, often presenting in childhood with alterations in skin pigmentation, skeletal abnormalities, and benign tumours called neurofibromas [6–8]. An often overlooked but significant symptom is neuropathic pain, which affects 22–70% of NF1 patients and has a profound impact on their health-related quality of life [9–16]. Despite its prevalence, the mechanisms underlying NF1-associated pain remain poorly understood [9, 17]. Pain can result either from tumour-induced nerve compression or from tumour-independent mechanisms, such as altered ion channel expression or neuropeptide release, leading to increased excitability in dorsal root ganglia (DRG) neurons [3, 4].

This highlights the importance of the DRG in the pathophysiology of neuropathic pain, particularly in NF1 but also in many other pain syndromes [18]. Recently, high-resolution magnetic resonance imaging (MRI) has been optimised to assess the human DRG at the organ level *in vivo*. While Godel et al analysed DRG volume and were able to differentiate neurofibromatosis type 2 (NF2)-associated schwannomatosis from schwannomatosis (formerly called neurofibromatosis type 3) using this quantitative DRG feature [19], to our knowledge, there is currently no *in vivo* MRI study of human DRG in NF1 patients.

In addition to assessing macrostructural changes, quantitative MRI can detect microstructural changes in the DRG by analysing signal intensities within specific tissue compartments [20–24]. *In vivo* studies of human DRG in NF1 may contribute to the understanding of the pathophysiology of pain in NF1, particularly in attempting to distinguish disease-specific from pain-specific changes in NF1. Furthermore, the identification of non-invasive MRI biomarkers has the potential to inform clinical decision-making and serve as endpoints for future longitudinal studies. This exploratory investigation of the underlying microstructural changes also has the broader goal of assessing individual pain risk and monitoring therapy as part of personalised pain medicine in NF1 and beyond.

In this study, we analysed quantitative lumbosacral DRG MRI in deeply phenotyped NF1 patients and healthy controls *in vivo*. The aims of our exploratory observational study were to objectify potential DRG changes in NF1 compared to healthy controls and to detect possible macro- or microstructural DRG correlates in NF1-associated neuropathy.

## Methods

### Study population

From May 2022 to November 2023, 48 consecutive subjects were included in this study. All of them provided written informed consent beforehand. An ethics approval was obtained from the responsible Ethics Committee of the University of Würzburg (Institutional Review Board #136/20). The study was conducted in accordance with the Declaration of Helsinki.

**Fig. 1:**
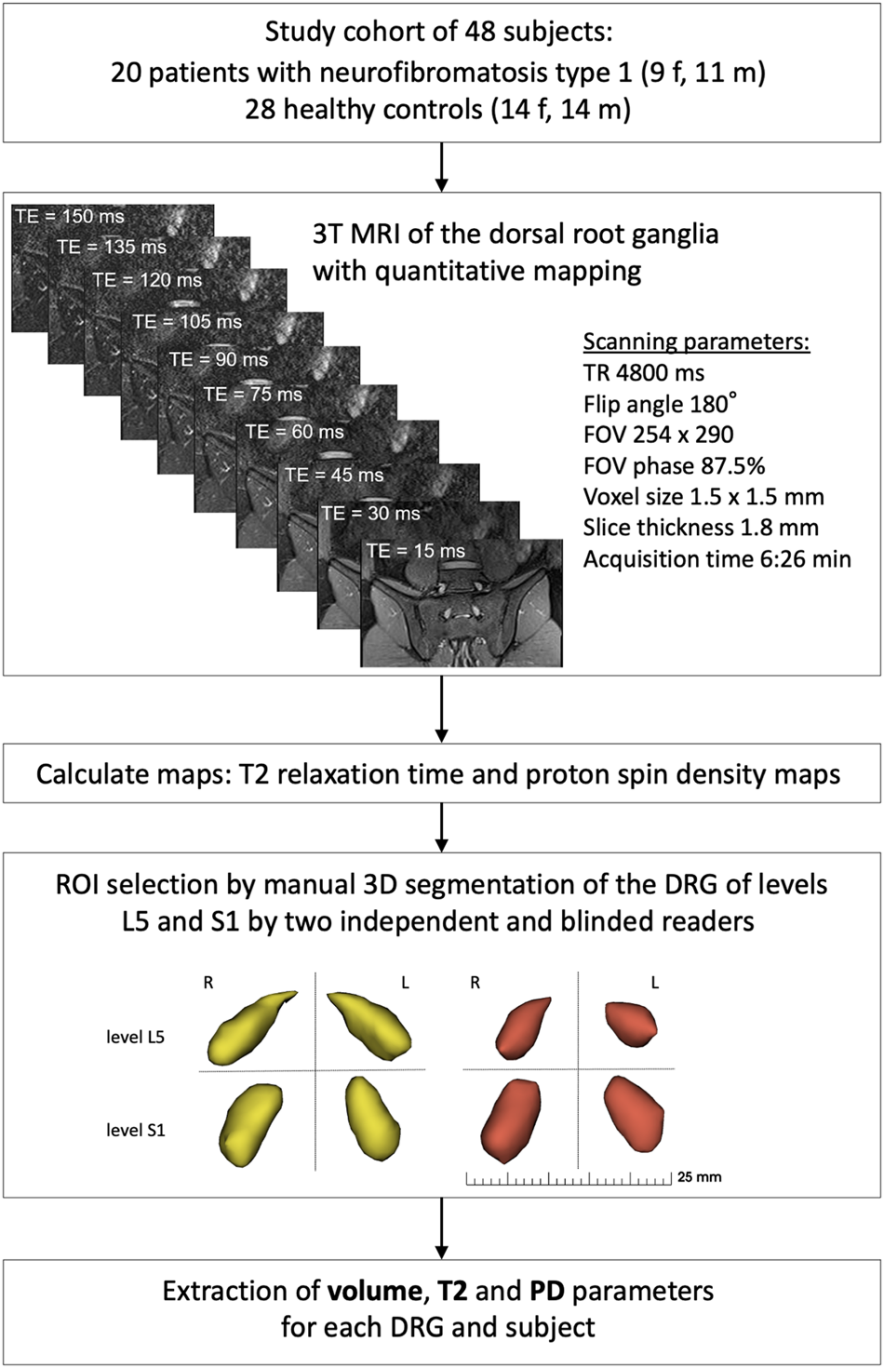
Flowchart of the study design. DRG, Dorsal root ganglion/ganglia; FOV, Field of view; L5, Lumbar level 5; MRI, Magnetic resonance imaging; PD, Proton density; ROI, Region of interest; S1, Sacral level 1; T2, T2 relaxation time; TR, Repetition time

The study cohort was composed as follows: 20 consecutive NF1 patients were included in the study and divided into groups according to the presence of neuropathic pain (NF1p, *n* = 8) or absence of neuropathic pain (NF1n, *n* = 12). The allocation to one of the two groups was made by two neurosurgeons with experience in the clinical management of NF1 patients (E.M. and C.M.) based on medical history and clinical examination (Supplementary Table S1). The inclusion criteria for the pain group were an average pain score of ≥ 3 on the visual analogue 0–10 scale over the previous 10 weeks. All pain cases included were chronic, had been present for more than one year, and were in stable condition at the time of the study. Other causes of neuropathic pain and polyneuropathy were excluded by detailed medical history and electrophysiological studies. In addition, severe psychiatric disorders were predefined exclusion criteria. Further exclusion criteria for subjects were minority, pregnancy, ongoing infectious disease, substance abuse, history of back surgery, and insufficient physical fitness to participate in the studies. Twenty-eight consecutive healthy subjects without a pain syndrome or any form of neurological or metabolic disease served as the healthy control group. The study design is summarised in Fig. 1.

### MRI protocol and image postprocessing

All participants underwent imaging of the lumbosacral plexus at the same 3-T scanner (PRISMAfit, Siemens Healthineers, Erlangen, Germany) at the Department of Neuroradiology of the University Hospital Würzburg.

The acquisition protocol included a multiecho spin-echo sequence for quantitative evaluation, with the following technical parameters: echo train length 10; Δ echo time range 15 ms; repetition time: 4,800 ms; flip angle 180°; in plane resolution 1.5 × 1.5 mm; slice thickness 1.8 mm; interslice gap 1.8 mm). The slice orientation was aligned paracoronal through the DRG of the lumbar 5 (L5) and sacral 1 (S1) levels.

Image postprocessing was performed via MATLAB (The MathWorks, Inc., 2024: MATLAB, version R2024a Update 3, retrieved from https://www.mathworks.com/) using a custom-built script [25, 26]. Maps were fitted for T2 relaxation time and initial magnitude of magnetisation as an indicator of proton spin density (PD), taking into account B_1_ field inhomogeneities.

Quality control was performed, including checking the statistics of the fitting routine for plausibility and possible outliers. In addition, once the quantitative maps had been generated, a visual check was carried out to check for any artefacts or irregularities in the maps. To quantify DRG PD, the initial magnetisation magnitude (equilibrium magnetisation) of the DRG was normalised to the internal reference of the cerebrospinal fluid.

### Image analysis and feature extraction

Image analysis was performed by two independent readers with 5 and 6 years of experience in neuroimaging (S.W. and M.S.). The two readers were blinded to each other, to diagnosis, and to demographic characteristics of the analysed subjects. The two readers independently performed three-dimensional selection of the volume of interest by manual segmentation of the DRG of levels L5 and S1 via 3D Slicer (The Slicer Community 2024: 3D Slicer, version 5.6.2, retrieved from: https://www.slicer.org/). In this way, a total of 192 DRGs were analysed (48 subjects × 4 DRGs each). Subsequently, a three-dimensional model of every DRG was created, and DRG volume was calculated (number of voxels within the volume of interest x voxel size). From the segmentations, mask images were generated and multiplied by the calculated T2 and PD maps. From this, the features of DRG T2 and DRG PD were extracted for each DRG. DRG features were calculated independently for both readers to estimate the inter-rater reliability. For the statistical analyses, the mean of the values from the two readers was calculated.

### Statistical analysis

Statistical analyses and visualisations were performed with R (The R Foundation for Statistical Computing 2024: R: A language for Data Analysis and Graphics, version 4.4.1, retrieved from: https://www.r-project.org/) and GraphPad Prism (GraphPad Prism version 9.4.0, GraphPad Software).

For descriptive statistics, all data is given as median (interquartile range) or number (*n*) (percentage), respectively. For comparisons of paired or unpaired groups, the Mann–Whitney *U*-test or the Wilcoxon signed-rank test was used for continuous variables. For the dichotomous variable sex, the χ^2^ test or Fisher’s exact test was used. Spearman correlation coefficient ρ was calculated between demographic parameters and DRG features to estimate their impact. The inter-rater reliability of DRG features was estimated via the intraclass correlation coefficient (two-way, agreement). Testing each DRG feature as a discriminator between pain and no pain phenotype in NF1 was performed using receiver operating characteristic analysis and logistic regression analysis. Its area under the curve (AUC) was calculated to estimate its performance. An appropriate cutoff value for DRG PD to discriminate between a painful and a nonpainful NF1 phenotype was determined based on the Youden index (= sensitivity + specificity - 1). Model fit was assessed using McFadden’s *R*^2^, indicating explanatory power, and the Akaike Information Criterion, reflecting model quality with a penalty for complexity. Lower AIC values and higher McFadden’s *R*^2^ suggest better model performance. Probability values of ≤ 0.05 were estimated as statistically significant.

Due to the exploratory nature of the study and the fact that data for each subject were aggregated as means of DRG characteristics per subject, correction for multiple comparisons was not applied.

## Results

### Study cohort

The demographic characteristics of the study cohort are presented in Table 1. In summary, there were no differences between the healthy control cohort and the NF1 patient cohort regarding sex (*p* = 0.961), age (*p* = 0.104), height (*p* = 0.414), weight (*p* = 0.374), or body mass index (*p* = 0.554).

**Table 1:** Demographic and DRG characteristics for the cohorts of healthy controls and patients with NF1

	Healthy controls (*n* = 28)	NF1 patients (*n* = 20)	
Parameter	Median (IQR) or *n* (%)	Median (IQR) or *n* (%)	*p*-value
Age [years]	40.5 (26.5)	31.0 (21.3)	0.104
Sex			0.961
Female	14 (50.0%)	9 (45.0%)	
Male	14 (50.0%)	11 (55.0%)	
Body height [cm]	174.0 (5.5)	170.0 (9.3)	0.414
Body weight [kg]	71.5 (19.0)	69.0 (17.0)	0.374
Body mass index [kg/m^2^]	23.6 (5.6)	23.8 (3.5)	0.554
DRG volume [mm^3^]			
L5	456.4 (189.5)	1,420.1 (1,040.3)	< 0.001
S1	777.1 (216.5)	2,127.2 (2,471.3)	< 0.001
Mean of L5 and S1	628.4 (243.1)	1,736.5 (1,604.5)	< 0.001
DRG T2 [ms]			
L5	95.4 (14.0)	107.4 (24.3)	0.036
S1	93.5 (20.1)	118.1 (35.4)	0.005
Mean of L5 and S1	96.2 (16.6)	118.2 (29.5)	0.009
DRG PD [a.u.]			
L5	0.824 (0.133)	0.883 (0.072)	0.067
S1	0.836 (0.091)	0.870 (0.150)	0.021
Mean of L5 and S1	0.833 (0.103)	0.903 (0.113)	0.007

Mann–Whitney *U*-test or χ^2^ test was used for group comparisons

*a.u.* Arbitrary units, *DRG* Dorsal root ganglion, *IQR* Interquartile range, *L5* Lumbar level 5, *NF1* Neurofibromatosis type 1, *S1* Sacral level 1

### Inter-rater reliability of DRG MRI features

The inter-rater reliability was calculated based on all 192 individual DRG masks per reader. The inter-rater intraclass correlation coefficient (two-way, agreement) was 0.94 (95% confidence interval 0.87–0.97) for DRG volume, 0.70 (0.60–0.78) for DRG T2, and 0.98 (0.94–0.99) for DRG PD.

### Alterations of DRG MRI features in NF1

**Fig. 2:**
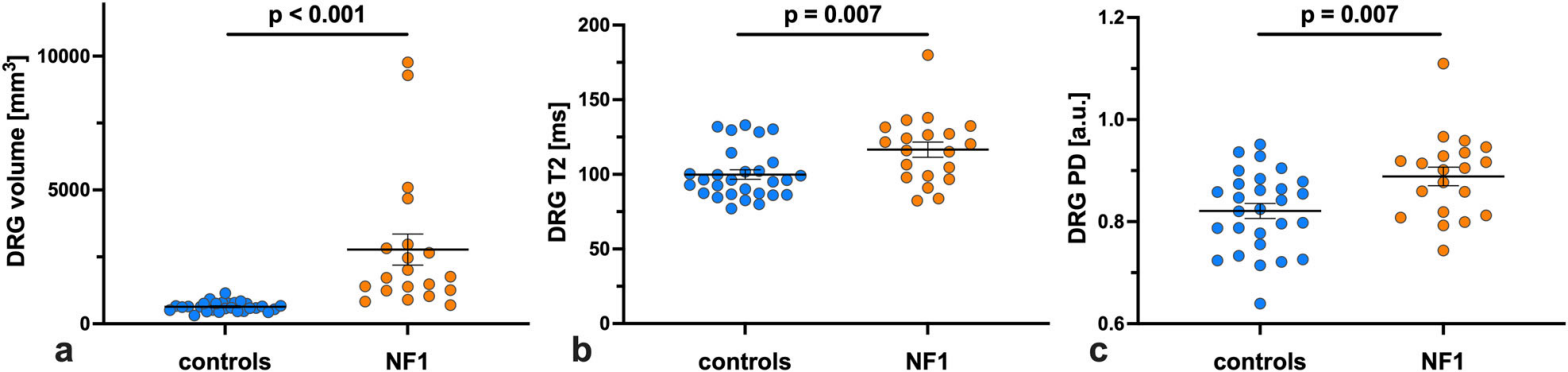
DRG from patients with NF1 (*n* = 20) showed a significant increase in volume (+176.3%; median 1,736.5 *versus* 628.4 mm³, *p* < 0.001) (**a**), T2 relaxation time (+22.9%; median 118.2 *versus* 96.2 ms, *p* = 0.007) (**b**), and PD (+8.4%; median 0.903 *versus* 0.833 a.u., *p* = 0.007) (**c**) compared to healthy controls (*n* = 28). DRG, Dorsal root ganglion/ganglia; NF1, Neurofibromatosis type 1; PD, Proton density; T2, T2 relaxation time

Differences were found regarding all three analysed DRG imaging features (Table 1) between NF1 patients and healthy controls. The DRG of NF1 patients were larger (median 1,736.5 *versus* 628.4 mm³, *p* < 0.001; Fig. 2a). This was true for both L5 and S1 levels (*p* < 0.001, each). The DRG at the S1 level were larger than those at the L5 level, both in healthy controls (median 777.1 *versus* 456.4 mm³, *p* < 0.001) and in NF1 patients (median 2,127.2 *versus* 1,420.1 mm³, *p* = 0.008). There were no differences in volumes between corresponding left and right DRG in healthy controls (median 619.7 *versus* 631.3 mm³, *p* = 0.168) or in NF1 patients (median 1,872.1 *versus* 1,689.8 mm³, *p* = 0.756). No differences in DRG volume were observed between male and female subjects, neither in the healthy control cohort (median 628.0 *versus* 616.0 mm³, *p* = 0.735) nor in the NF1 patient cohort (median 2,458.0 *versus* 1,477.0 mm³, *p* = 0.152). There was no correlation between DRG volume and age (healthy controls: ϱ = 0.11, *p* = 0.572; NF1: ϱ = -0.10, *p* = 0.660) or weight (healthy controls: ϱ = 0.26, *p* = 0.175; NF1: ϱ = -0.03, *p* = 0.890).

### DRG T2 relaxation time

The DRG T2 relaxation time in NF1 patients was longer than in healthy controls (median 118.2 *versus* 96.2 ms, *p* = 0.007; Fig. 2b). This was true for both L5 (median 107.4 *versus* 95.4 ms, *p* = 0.036) and S1 levels (median 118.1 *versus* 93.5 ms, *p* = 0.005). There was no difference in DRG T2 between S1 and L5 levels in healthy controls (median 93.5 *versus* 95.4 ms, *p* = 0.639), but it was higher for S1 in NF1 patients (median 118.1 *versus* 107.4 ms, *p* = 0.020). No differences in DRG T2 were observed between corresponding left and right DRG in healthy controls (median 97.1 *versus* 92.9 ms, *p* = 0.855), whereas a difference was found in NF1 patients (mean 119.7 *versus* 113.4 ms, *p* = 0.015) despite the same median (median 115.6 *versus* 115.6 ms). No differences in DRG T2 were found between male and female subjects, neither in the healthy control cohort (median 94.0 *versus* 99.4 ms, *p* = 0.376) nor in the NF1 patient cohort (median 120.0 *versus* 107.0 ms, *p* = 0.656). There was no correlation between DRG T2 and age (healthy controls: ϱ = -0.32, *p* = 0.097; NF1: ϱ = 0.01, *p* = 0.791) or weight (healthy controls: ϱ = -0.21, *p* = 0.274; NF1: ϱ = -0.06, *p* = 0.890).

### DRG PD

The DRG PD in NF1 patients was higher than in healthy controls (median 0.903 *versus* 0.833 arbitrary units (a.u.), *p* = 0.007; Fig. 2c). This was also true for the S1 level when considered in isolation (median 0.870 *versus* 0.836 a.u., *p* = 0.021), but not for the L5 level (median 0.883 *versus* 0.824 a.u., *p* = 0.067). There was no difference in DRG PD between S1 and L5 levels, neither in healthy controls (median 0.836 *versus* 0.824 a.u., *p* = 0.582) nor in NF1 patients (median 0.870 *versus* 0.883 a.u., *p* = 0.261). No differences in DRG PD were observed between corresponding left and right DRG in healthy controls (median 0.846 *versus* 0.822 a.u., *p* = 0.831) or in NF1 patients (median 0.894 *versus* 0.879 a.u., *p* = 0.064). No differences in DRG PD were found between male and female subjects, neither in the healthy control cohort (median 0.809 *versus* 0.856 a.u., *p* = 0.454) nor in the NF1 patient cohort (median 0.914 *versus* 0.858 a.u., *p* = 0.175). There was no correlation between DRG PD and age (healthy controls: ϱ = 0.26, *p* = 0.184; NF1: ϱ = 0.002, *p* = 0.992) or weight (healthy controls: ρ = 0.26, *p* = 0.176; NF1: ρ = 0.12, *p* = 0.622).

### Increase of DRG PD in NF1 patients with neuropathic pain

**Fig. 3:**
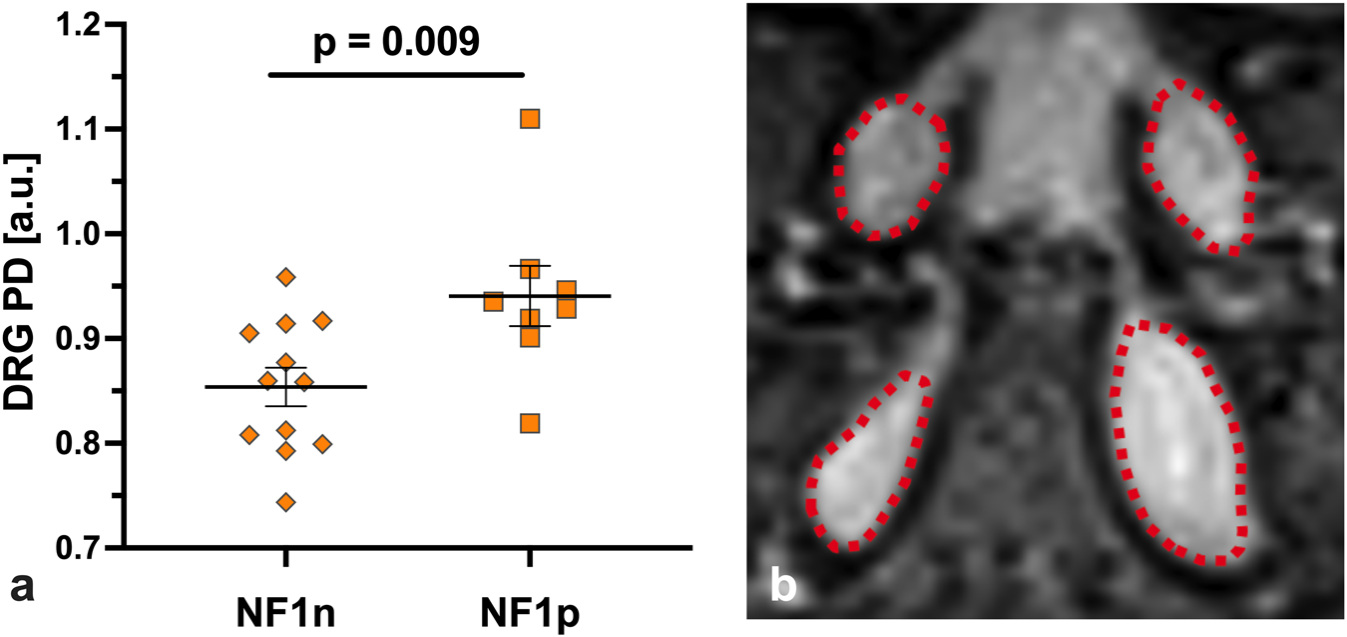
PD of DRG in patients with neurofibromatosis type 1 with a pain phenotype (NF1p; *n* = 8) significantly increased compared to patients with neurofibromatosis type 1 without pain phenotype (NF1n; *n* = 12), median 0.932 *versus* 0.859 a.u., *p* = 0.009 (**a**). Example of PD-weighted contrast, the contour of the DRGs is marked with red dotted lines (**b**). DRG, Dorsal root ganglion/ganglia; NF1n, Neurofibromatosis type 1 with no-pain phenotype; NF1p, Neurofibromatosis type 1 with pain phenotype; PD, Proton density

In 8 of the 20 NF1 patients (40.0%), the diagnosis of NF-associated painful neuropathy was confirmed. The NF1 cohort was subdivided into groups NF1p and NF1n based on this diagnosis, and the subgroups were re-evaluated regarding the analysed DRG imaging features (Table 2). Differences were found in DRG PD, showing an increase for the NF1p subcohort (median 0.932 *versus* 0.859 a.u., *p* = 0.009; Fig. 3). When examining individual levels separately, this result was only reproducible for the S1 level. Regarding the other DRG imaging features, there were trends observed (increase of DRG volume and DRG T2 for NF1p), but no significant differences were identified.

**Table 2:** Demographic and DRG characteristics for the subgroups of patients with NF1 with and without painful neuropathy

	NF1n (*n* = 12)	NF1p (*n* = 8)	
Parameter	median (IQR) or *n* (%)	median (IQR) or *n* (%)	*p*-value
Age [years]	31.0 (10.5)	33.0 (24.8)	0.847
Sex			0.670
Female	6 (50.0%)	3 (37.5%)	
Male	6 (50.0%)	5 (62.5%)	
Body height [cm]	170.0 (5.8)	175.5 (11.5)	0.215
Body weight [kg]	67.5 (12.8)	72.5 (19.8)	0.463
Body mass index [kg/m^2^]	23.8 (3.0)	23.5 (4.1)	0.910
DRG volume [mm^3^]			
L5	1,345.7 (792.4)	1,869.2 (5,189.8)	0.263
S1	1,842.9 (1,004.6)	3,884.1 (3,687.3)	0.082
Mean of L5 and S1	1,557.9 (936.8)	2,894.9 (4,411.1)	0.115
DRG T2 [ms]			
L5	105.3 (24.3)	111.9 (33.5)	0.305
S1	104.9 (28.1)	136.6 (32.8)	0.114
Mean of L5 and S1	110.3 (25.2)	125.7 (20.8)	0.135
DRG PD [a.u.]			
L5	0.883 (0.100)	0.879 (0.077)	0.427
S1	0.843 (0.067)	0.985 (0.079)	0.020
Mean of L5 and S1	0.859 (0.102)	0.932 (0.037)	0.009

Mann–Whitney *U-*test or χ^2^ test was used for group comparisons

*a.u.* Arbitrary units, *DRG* Dorsal root ganglion, *IQR* Interquartile range, *L5* Lumbar level 5, *NF1n* Neurofibromatosis type 1 without painful neuropathy, *NF1p* Neurofibromatosis type 1 with painful neuropathy, *S1* Sacral level 1

### Discriminators between pain and non-pain phenotype in NF1

**Fig. 4:**
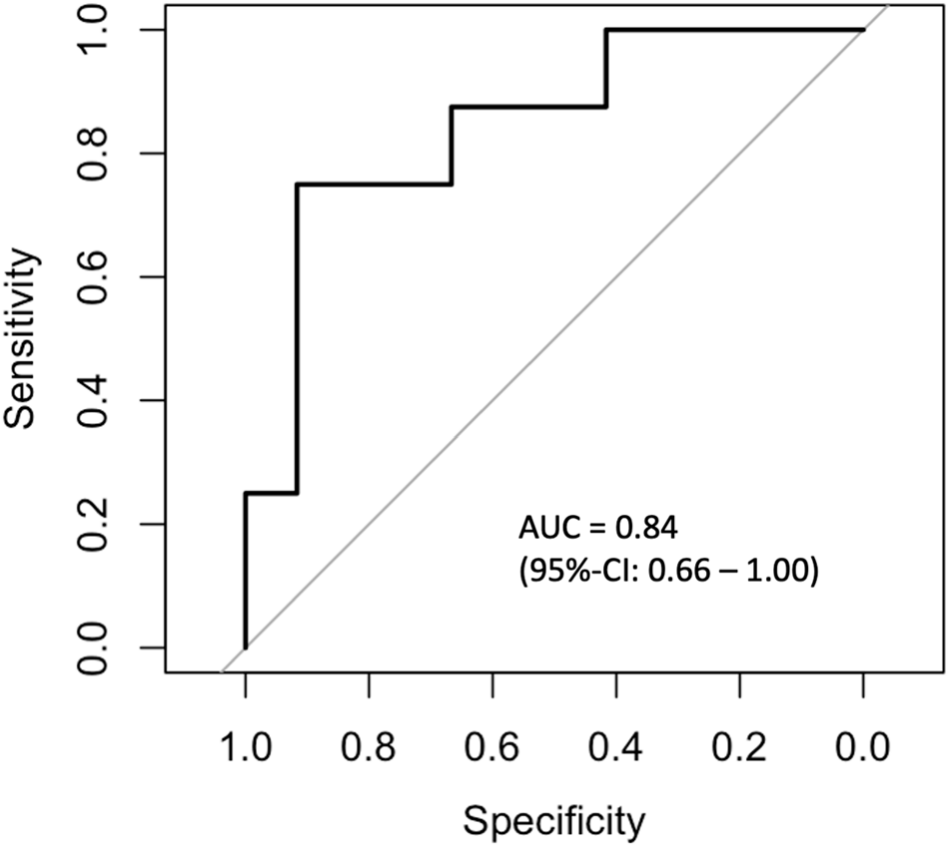
Receiver operating characteristic analysis for the parameter DRG PD to discriminate between NF1 patients with and without pain phenotype. The logistic regression model showed a significant improvement with DRG PD (*p* = 0.008), moderate explanatory power (McFadden *R*^2^ = 0.26), and good discriminatory ability (AUC = 0.84, 95% confidence interval 0.66–1.00). DRG, Dorsal root ganglion/ganglia; NF1, Neurofibromatosis type 1; PD, Proton spin density

For discriminating between pain and non-pain phenotype in NF1 patients, the DRG PD parameter (AUC = 0.84; Fig. 4) was superior to DRG volume (AUC = 0.72) and DRG T2 (AUC = 0.71) when performing receiver operating characteristic analyses for each parameter (Supplementary Table S2). The corresponding 95% confidence interval of the AUC of DRG PD ranged from 0.66 to 1.00, indicating a good discriminatory ability of the model. For the predictor DRG PD, the intercept of the univariate logistic regression model was estimated to be β = -19.73 with a standard error of 10.02, giving a *z*-statistic of -1.97 and a corresponding *p*-value of 0.049. The predictor DRG PD had an estimated coefficient (β) of 21.59 with a standard error of 11.10, a *z*-statistic of 1.95 and a *p*-value of 0.052. In terms of model fit, McFadden’s *R*^2^ was 0.26, indicating moderate explanatory power. The likelihood ratio test yielded a *p*-value of 0.008, indicating that the inclusion of DRG PD significantly improved the model. The Akaike Information Criterion was 23.97, providing a measure of model quality, with lower values indicating better fit. The resulting DRG PD threshold for discriminating between pain and non-pain NF1 phenotypes, with thresholds based on the Youden index to find the best values for maximising both sensitivity and specificity, was 0.918 a.u., giving a sensitivity of 0.75 and a specificity of 0.92 for discriminating NF1p from NF1n.

## Discussion

Our study shows differences in DRG MRI characteristics between NF1 patients and healthy controls, with all three features analysed (DRG volume, T2 relaxation time, and PD) showing distinct changes. The main findings are DRG enlargement and increased T2 relaxation time in NF1 patients. Interestingly, the subgroup of NF1 patients with neuropathic pain could be distinguished from those without pain based on DRG PD, highlighting the pathophysiological relevance of this parameter. To the best of the authors’ knowledge, no comparable study has been performed on human DRG in NF1 patients.

Reliable and comparable analysis of the DRG requires both a dedicated MRI protocol and sophisticated post-processing, including correction for B_1_ field inhomogeneities to minimise artefacts and measurement errors [27, 28]. The DRG volumes and T2 values in our healthy controls were within the expected ranges from previous studies [21]. Minor differences may be due to different acquisition techniques; for example, unlike other studies, our analysis used three-dimensional masks for each DRG. To quantify PD, DRG equilibrium magnetisation values were normalised to reference values of cerebrospinal fluid from the equilibrium magnetisation map, which provides information on the initial tissue magnetisation [29]. From a methodological point of view, it is mandatory that each individual reference value of cerebrospinal fluid for signal correction of each individual DRG is taken at the same distance from the receiving coil to account for the coil. Voxel-wise DRG segmentation was performed manually by two independent, blinded raters, with a very good inter-rater intraclass correlation coefficient for DRG volume and PD and a good inter-rater intraclass correlation coefficient for T2 [30].

Our first major finding was the correlation of NF1 with DRG enlargement. Interestingly, a similar study in NF2 patients showed that DRGs were also enlarged in patients with NF2-associated schwannomatosis compared with patients with schwannomatosis (formerly called neurofibromatosis type 3), likely due to schwannomas of the lumbosacral plexus originating from or involving the DRG [19]. Preclinical studies in NF2 mouse models have demonstrated DRG enlargement at all vertebral levels, with histological analysis revealing Schwann cell proliferation as a possible explanation [31]. Finally, clustered areas of Schwann cell proliferation have also been identified in humans as a correlate of DRG enlargement [32]. Histological analysis of the DRG of an NF2 patient showed that they were massively infiltrated by schwannoma tissue, with the regular architecture of a more central, nerve fibre-rich area and a more peripheral, cell body-rich area completely disrupted, indicating a remodelling of the DRG [32, 33]. Although neurofibromas predominate in NF1, concepts from NF2 studies can be considered for DRG enlargement in NF1 [34]. An increased spatial affinity of neurofibromas to the DRG has been described in mouse models, and a case report of an NF1 patient confirmed tumour infiltration of the DRG [31, 35]. Thus, DRG enlargement in NF1 is likely to reflect tumour-related structural changes caused by diffuse tumour infiltration.

Beyond volumetric changes, DRG MRI allows quantitative analysis of tissue microstructure through T2 relaxometry [21]. T2 relaxometry is a key component of quantitative MRI neurography, as it reflects the molecular and cellular composition of the tissue [23, 36].

Our second major finding was the increase of the DRG T2 relaxation time in NF1 patients, suggesting disruption of DRG architecture due to tumour manifestations. This is in line with findings in glioma patients, where increased T2 relaxation times were associated with increased tissue infiltration and disrupted architecture, particularly in isocitrate dehydrogenase mutant gliomas [37, 38]. Interestingly, while DRG volume and T2 relaxation time correlated with the presence of NF1, they did not discriminate between pain phenotypes within the NF1 cohort.

Our third major finding was the discrimination between painful and non-painful NF1 phenotypes by DRG proton spin density, with higher values being associated with a painful NF1 phenotype. This finding is clinically relevant as it provides a potential biomarker for pain phenotyping in NF1, addressing a long-standing challenge in the objective measurement of pain in general. Importantly, this increase in PD was independent of the observed DRG enlargement, highlighting its unique diagnostic value.

At the molecular level, an increased PD indicates an increase in the total measurable water content within the DRG, which differs from T2 relaxation time, as the latter specifically reflects the free water content. [23, 36]. The constellation of increased PD without a corresponding increase in T2 relaxation time, as observed in painful NF1, suggests an accumulation of water bound to macromolecules or restricted by structural components such as myelin sheaths. This pattern argues against acute inflammation or oedema and suggests microstructural or molecular changes in the DRG. These findings are consistent with models of small fibre neuropathy, which is often associated with pain, pruritus and paraesthesia—symptoms commonly reported in NF1 patients [3, 39, 40].

Peripheral neuropathy in NF1 may result from tumour-related nerve compression or infiltration, as well as independent mechanisms such as small fibre neuropathy. Animal models have implicated dysregulation of calcium and sodium channels in DRG neurons as contributing to altered pain signalling in NF1 [41]. While *in vivo* DRG MRI cannot directly detect ion channel dysfunction, it may provide a valuable means of quantifying the structural and molecular correlates of these changes at the organ level.

Previous studies have used T2 relaxometry to evaluate peripheral nerve changes in several conditions, including nerve compression due to disc herniation [42, 43], chronic inflammatory demyelinating polyradiculoneuropathy [44], diabetic neuropathy [45–48], amyloidotic polyneuropathy [49, 50], multiple sclerosis [51] and Fabry disease [52]. These studies underscore the role of macromolecular composition in shaping T2 signal intensity and highlight its diagnostic utility in distinguishing from other disease states such as acute inflammation, oedema and demyelination.

Our study has the following limitations.

Although the resolution of our images, and thus the segmentation, was sufficient to detect volume differences between the groups, and the acquisition parameters were consistent across subjects, the resolution was still slightly lower than in other of our dedicated morphometric DRG studies [53]. To minimise inter-rater variability and human bias in DRG segmentation, increase its accuracy and reproducibility, and enable robust and time-efficient processing of even larger study cohorts, future studies should consider automated DRG segmentation.

As NF1 is a rare disease, and our patient group was therefore relatively small, with only 20 patients, the validity of our results is limited, especially when the NF1 cohort is further divided into subcohorts with and without neuropathic pain. Although there were no demographic differences between the NF1 patient cohort and the healthy control cohort in this exploratory study, patients should be matched for age and sex in future clinical trials. In addition, although psychiatric disorders were an exclusion criterion for the study in order to keep the cohort as representative as possible, individual confounders such as pain medication, especially chronic pain medication, could affect individual pain perception and neuropathic changes and therefore influence DRG imaging parameters and their significance.

Furthermore, while our findings suggest that DRG imaging features reflect deeper microstructural and molecular changes, histological validation in NF1 models remains essential to clarify the relationship between DRG PD and neuropathic pain in NF1 and to confirm our observations. For future *in vivo* studies, in addition to the DRG features evaluated here, volume, T2 relaxometry and PD, other MR imaging features such as diffusion tensor imaging or magnetisation transfer ratio may be used to characterise possible changes in nerve fibre integrity, myelination or fibrotic changes of the DRG [54, 55].

In conclusion, our study provides novel insights into the structural and functional alterations of the DRG in NF1 patients. DRG enlargement coupled with increased T2 relaxation times and increased PD, underscores the complex pathophysiological changes associated with NF1 neuropathy. Our results suggest that DRG changes in NF1 quantified by DRG MRI may reflect deeper microstructural and molecular changes, which in turn may provide insights into the still unclear pathophysiology of neurofibromatosis neuropathy. These findings support DRG MRI as a promising biomarker for NF1 neuropathy and as a potential clinical endpoint for future trials.

## Supplementary information

**Additional file 1: Table S1.** Overview of the 20 consecutive Neurofibromatosis type 1 patients and their pain characteristics. Subgroup status according to presence of NF1-associated neuropathic pain. **Table S2.** Predictive models to discriminate between NF1 with and without painful neuropathy.

## Data Availability

The datasets used and/or analysed during the current study are available from the corresponding author on reasonable request.

## Abbreviations

a.u.: Arbitrary units
AUC: Area under the curve
DRG: Dorsal root ganglion/ganglia
L5: Lumbar level 5
MRI: Magnetic resonance imaging
NF1: Neurofibromatosis type 1
NF1n: Neurofibromatosis type 1 without neuropathic pain
NF1p: Neurofibromatosis type 1 with neuropathic pain
NF2: Neurofibromatosis type 2
PD: Proton density
S1: Sacral level 1
