# Supplementary material for: Quantitative MRI of dorsal root ganglion alterations in neurofibromatosis type 1 patients with or without pain
Source: Eur Radiol Exp. 2025 May 28;9:57. doi: 10.1186/s41747-025-00594-x (PMC12119455; doi:10.1186/s41747-025-00594-x)
Supplement: Supplementary file 1 — Additional file 1: Table S1. Overview of the 20 consecutive Neurofibromatosis type 1 patients and their pain characteristics. Subgroup status according to presence of NF1-associated neuropathic pain. Table S2. Predictive models to discriminate between NF1 with and without painful neuropathy. [file 41747_2025_594_MOESM1_ESM.pdf]

Quantitative MRI of dorsal root ganglion alterations in neurofibromatosis Type 1 patients with or without pain

ELECTRONIC SUPPLEMENTARY MATERIAL

Eur Radiol Exp (2025) Schindehütte M, Meller E, Kampf T, et al.

Table S1 Overview of the 20 consecutive Neurofibromatosis type 1 patients and their pain characteristics. Subgroup status according to presence of NF1-associated neuropathic pain

**Patient number**	**Sex, age** [years]	**Subgroup**	**Other pain syndromes**	**Analgesic medication**	**Pain localisation**
1	Female, 19	NF1n	Previously right groin pain associated with neurofibroma; pain resolution after surgery	Previously opioids (before surgery)	–
2	Male, 31	NF1n	None	None	–
3	Female, 35	NF1n	Complex regional pain syndrome	Antidepressant, anticonvulsant	–
4	Male, 24	NF1p	None	NSAIDs, anticonvulsant	Lumbalgia and left gonalgia
5	Male, 57	NF1p	None	Opioids, anticonvulsant	Cervical and abdominal pain
6	Female, 53	NF1n	None	None	–
7	Female, 28	NF1n	None	None	–
8	Female, 24	NF1p	None	THC	Right femoral pain
9	Male, 42	NF1p	None	THC, NSAIDs	Lumbalgia with bilateral lower limb involvement
10	Male, 18	NF1p	None	NSAIDs, opioids	Lumbalgia with bilateral lower limb involvement
11	Female, 44	NF1p	None	NSAIDs, antidepressant	Pelvic, upper limb, and lower limb pain
12	Male, 49	NF1n	None	None	–
13	Female, 60	NF1p	None	Opioids	Lumbalgia with lower limb involvement
14	Male, 31	NF1n	migraine	None	–
15	Male, 28	NF1n	None	None	–
16	Male, 18	NF1p	None	NSAIDs, anticonvulsant	Left pedal pain
17	Male, 30	NF1n	None	None	
18	Female, 22	NF1n	None	None	
19	Female, 58	NF1n	None	None	
20	Male, 31	NF1n	None	None	–

NF1n Neurofibromatosis type 1 without painful neuropathy, *NF1p* Neurofibromatosis type 1 with painful neuropathy, *NSAIDs* Non-steroidal anti-inflammatory drugs, *THC* Tetrahydrocannabinol.

Table S2 Predictive models to discriminate between NF1 with and without painful neuropathy

a. Univariate logistic regression model (volume)

Parameter	β	SE	z-statistic	*p*-value
(Intercept)	-1,64	0,86	-1,91	0,056
DRG volume	0,0005	0,0003	1,57	0,116

McFadden R^2 = 0.16, Likelihood-ratio-test p = 0.038, AIC = 26.63, AUC = 0.72 (95%-CI: 0.46 0.98)

b. Univariate logistic regression model (T2)

Parameter	β	SE	z-statistic	*p*-value
(Intercept)	-5,22	3,23	-1,62	0,106
DRG T2	0,04	0,03	1,52	0,129

McFadden's R^2 = 0.11, Likelihood-ratio-test p = 0.081, AIC = 27.88, AUC = 0.71 (95%-CI: 0.46 - 0.96)

c. Univariate logistic regression model (PD)

Parameter	β	SE	z-statistic	*p*-value
(Intercept)	-19,73	10,02	-1,97	0,049
DRG PD	21,59	11,10	1,95	0,052

McFadden's R^2 = 0.26, Likelihood-ratio-test p = 0.008, AIC = 23.97, AUC = 0.84 (95%-CI: 0.66 - 1.00)

d. Multivariate logistic regression model (volume, T2, PD)

Parameter	β	SE	z-statistic	*p*-value
(Intercept)	-19,3	12,3	-1,57	0,115
DRG volume	0,0001	0,0003	0,38	0,706
DRG T2	-0,02	0,05	-0,45	0,654
DRG PD	23,7	17,7	1,34	0,182

McFadden's R^2 = 0.27, Likelihood-ratio-test p = 0.061, AIC = 27.56, AUC = 0.85 (95%-CI: 0.68 - 1.00)

AIC Akaike Information Criterion, *AUC* Area under the curve, *DRG* Dorsal root ganglion, *NF1* Neurofibromatosis type 1, *PD* Proton density, *T2* T2 relaxation time.
